# Efficacy analysis of endovascular treatment for ruptured internal carotid artery pseudoaneurysm hemorrhage in patients with nasopharyngeal carcinoma after radiotherapy

**DOI:** 10.3389/fsurg.2024.1451570

**Published:** 2024-08-20

**Authors:** Zhipeng Lin, Xugong Zou, Yuan Chen, Xiaoqun Li, Jian Zhang, Dabei Huang

**Affiliations:** Department of Interventional Medicine, Zhongshan People’s Hospital, Zhongshan, Guangdong, China

**Keywords:** nasopharyngeal carcinoma, radiotherapy, internal carotid artery pseudoaneurysms, stent graft, pseudoaneurysm

## Abstract

**Background:**

This study aims to evaluate the efficacy and complications of endovascular treatment for hemorrhage caused by ruptured internal carotid artery pseudoaneurysms following radiotherapy in nasopharyngeal carcinoma (NPC) patients.

**Methods:**

This study retrospectively analyzed NPC patients who underwent endovascular treatment for ruptured internal carotid artery pseudoaneurysm hemorrhage after radiotherapy at Zhongshan People's Hospital from January 2016 to December 2022. The study aims to assess the postoperative hemostasis rate, postoperative rebleeding rate, complication rate, and 1-year postoperative survival rate.

**Results:**

During the study period, 36 patients underwent endovascular treatment, of which 24 patients underwent embolization of the internal carotid artery and 12 patients underwent stenting of the internal carotid artery. The procedure success rate was 100%. The rebleeding rate at 1 year after the procedure was 5.6% (2/36, one patient with stent placement and one patient with coil embolization), and the complication rate was 11.1% (4/36, four patients with coil embolization patients). Two patients developed large-area cerebral infarction after the procedure, and two patients had different degrees of neurological impairment after the procedure. The 1-year survival rate was 91.7% (33/36).

**Conclusion:**

Ruptured internal carotid artery pseudoaneurysm hemorrhage after radiotherapy is rare but life-threatening. Endovascular treatment with coil occlusion or stenting reconstruction of the internal carotid artery provides immediate hemostasis and elimination of the pseudoaneurysm with a low rate of recurrence, which may be effective in reducing patient mortality.

## 1 Introduction

Nasopharyngeal carcinoma (NPC) is the most common head and neck cancer in southern China (Guangdong, Fujian, Hunan, etc.) and Southeast Asia, with an incidence between 15 and 50 cases per 100,000 (1). Due to the deep and complex anatomy of the nasopharynx, radiotherapy remains the mainstay of curative treatment for NPC (2). Several complications can arise after radiotherapy for NPC, including nasopharyngeal hemorrhage. Ruptured internal carotid artery pseudoaneurysm hemorrhage after radiotherapy for NPC is a dangerous and fatal complication. Due to the deep position of the internal carotid artery, the effect of nasal cavity compression for hemostasis is often unsatisfactory (3). Surgical ligation is also not an appropriate treatment option due to the usual presence of lesion infection, large collateral vessels, and a high risk of surgical complications (4). Bypass grafting, radial artery high-flow bypass, and internal carotid artery pretreatment combined with endoscopic nasopharyngectomy offer new approaches for the surgical treatment of nasopharyngeal carcinoma with ruptured internal carotid artery pseudoaneurysms. However, these methods are not ideal for patients experiencing acute, life-threatening hemorrhage (5). Studies have shown that internal carotid artery occlusion is feasible if the balloon occlusion test shows adequate collateral circulation (6, 7). With the development of interventional therapy technology, an increasing number of patients are receiving interventional therapy. There are few reports on the interventional treatment of internal carotid artery pseudoaneurysm hemorrhage after radiotherapy for NPC. Therefore, this retrospective study aimed to evaluate the safety, efficacy, and complications of interventional treatment for ruptured internal carotid artery pseudoaneurysm hemorrhage in NPC patients after radiotherapy.

## 2 Methods

### 2.1 Inormal information

A total of 36 urgent patients with ruptured pseudoaneurysms of the internal carotid artery after radiotherapy for NPC were included at Zhongshan People’s Hospital from January 2016 to December 2022 for endovascular treatment.

The inclusion criteria included the following: (1) pathologically diagnosed nasopharyngeal carcinoma with a history of radiotherapy; (2) imaging examinations showing internal carotid artery pseudoaneurysm; and (3) single nasal bleeding >100 ml or continuous nasal bleeding >300 ml, with poor efficacy of anterior nasal packing. The exclusion criteria included the following: (1) severe insufficiency of heart, lung, and renal function and (2) internal carotid artery pseudoaneurysm bleeding caused by other reasons.

This study was approved by the Ethics Committee of Zhongshan People's Hospital (approval number 2024-050), Guangdong, China, and was conducted in accordance with the Declaration of Helsinki. Written informed consent was obtained from each patient.

### 2.2 Preoperative preparation

Before intervention treatment, relevant laboratory tests should be perfected, including blood routine tests, liver function tests, renal function tests, cardiac function tests, coagulation function tests, and computed tomography angiography of the carotid artery. Simultaneously, anterior nasal packing, active rehydration, and blood transfusion should be performed to correct hemorrhagic shock.

### 2.3 Interventional therapy

All procedures were performed in the interventional operating room by an experienced interventionalist. The patient is placed in the supine position (if the patient experiences nasopharyngeal bleeding, have the patient turn their head to one side to expel the secretions, preventing aspiration and potential suffocation. In the absence of nasopharyngeal bleeding, keep the patient's head facing forward to facilitate DSA imaging). Upper airway patency was maintained (tracheal intubation or tracheotomy if necessary), and routine ECG and blood oxygen saturation tests were conducted. First, the right femoral artery was punctured by the Seldinger technique, and a 6F catheter sheath (Terumo, Tokyo, Japan) was inserted. Next, a 5F vertebral artery catheter was selectively inserted into the bilateral common carotid arteries, external carotid arteries, and internal carotid arteries for arteriography. The location of the internal carotid artery pseudoaneurysm was determined according to the angiography. Based on the grading criteria of the American Society of Interventional and Therapeutic Neuroradiology/Society of Interventional Radiology (ASITN/SIR), for patients with nasopharyngeal carcinoma and internal carotid artery pseudoaneurysm rupture bleeding classified as ASITN/SIR 0-2 or ASITN/SIR 3 without compensation, our first treatment choice is the implantation of a covered stent in the lesion area of the internal carotid artery. If collateral circulation is well compensated, the internal carotid artery is occluded immediately with a coil. Following embolization, another angiogram was performed to assess its effectiveness. After stabilization, the femoral artery sheath was removed. Nasopharyngeal tamponade was removed within 48 h postoperatively, provided there was no nasal bleeding. For patients with a positive balloon occlusion test and not eligible for stent implantation, the subsequent treatment options were as follows: (1) conservative medical treatment; (2) common carotid/internal carotid artery ligation; (3) internal carotid artery coil occlusion; and (4) electrocoagulation for hemostasis via nasal endoscopy. After successful hemostasis, patients who received stenting were started on antiplatelet therapy with clopidogrel 75 mg daily and aspirin 100 mg daily, beginning on the third postoperative day, for at least 6 months. Coagulation routines were regularly reviewed, and the dosage was adjusted according to the results if necessary. Antiplatelet agents should be discontinued in the event of nasopharyngeal bleeding.

### 2.4 Evaluation of efficacy

Effective treatment was defined as the reduction or cessation of nasopharyngeal bleeding within 72 h of endovascular treatment, whereas ineffective treatment was defined as active bleeding that persisted despite endovascular treatment.

## 3 Results

This retrospective analysis included 42 patients diagnosed with internal carotid artery pseudoaneurysm hemorrhage after radiotherapy for NPC. However, six patients were excluded from the study because their families withdrew from the treatment after intraoperative angiography. The remaining 36 patients comprised 28 males and 8 females, with a mean age of 58.6 ± 8.9 years (37–72 years). All patients had undergone radiotherapy for NPC, specifically intensity-modulated radiation therapy (IMRT), with the radiation dose to the primary tumor ranging from 68 to 72 Gy, administered in 30–34 fractions. Additionally, 13 patients underwent re-irradiation with doses ranging from 45 to 60 Gy. The mean time from radiotherapy to pseudoaneurysm rupture was 7.2 ± 3.1 years (range, 2–15 years).

The patient presented with otorrhagia (*n* = 2) and epistaxis (*n* = 35). DSA angiography confirmed the presence of internal carotid artery pseudoaneurysms in 36 patients, of which 7 had pseudoaneurysms with contrast extravasation along the carotid sheaths. The lesions were located on the right side in 14 patients and on the left side in 22 patients. No external carotid arterial anomalies were detected in any of the patients.

**Figure 1:**
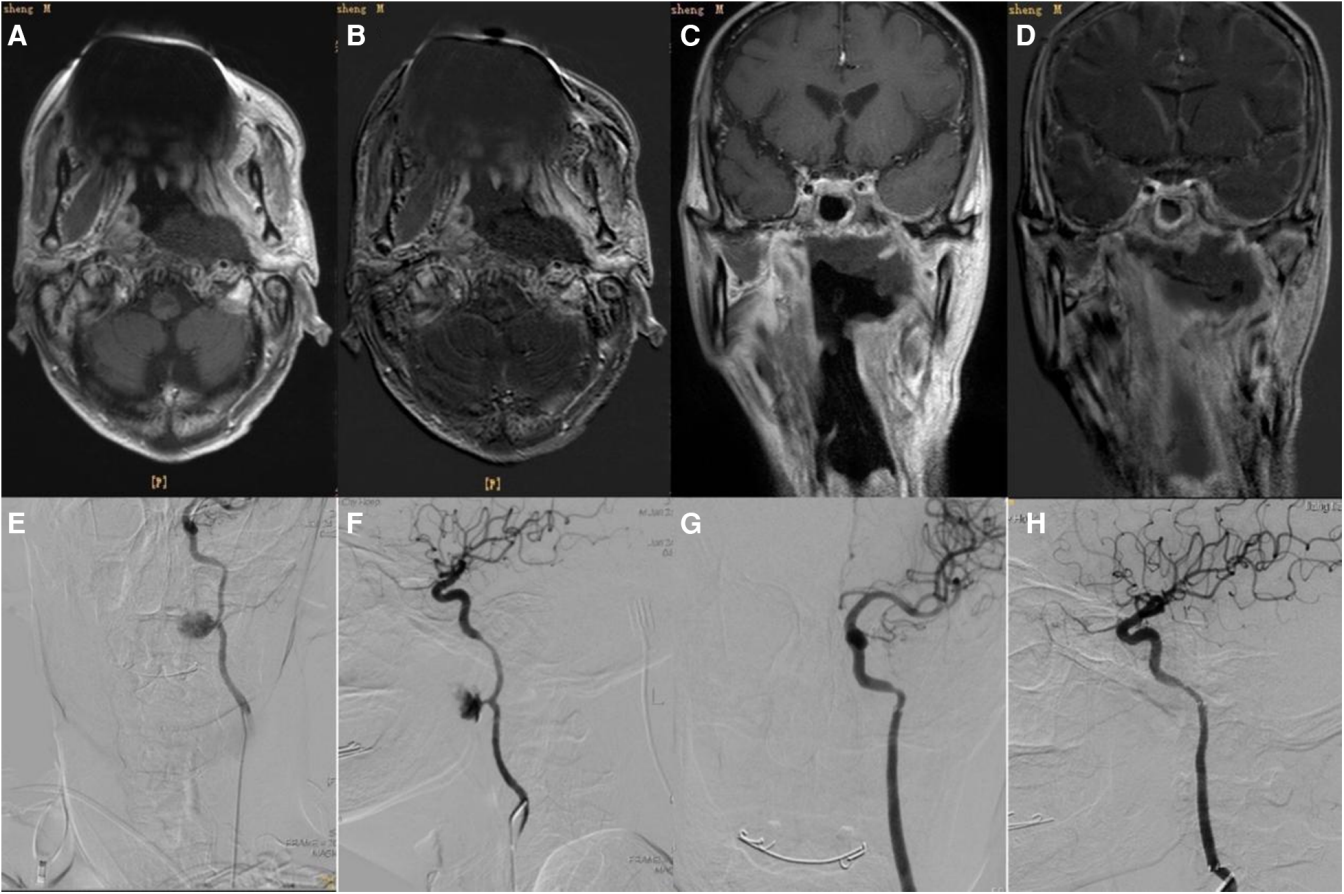
**(A)** Two weeks before interventional therapy, the patient's skull base, left carotid sheath, and foramen lacerum area showed patchy abnormal signals, which are considered to be fibrotic changes after radiotherapy. **(B)** No obvious enhancement was noted in the abnormal signal areas on the skull base, the left carotid sheath area, and the foramen lacerum area. **(C)** Coronal section. **(D)** Enhanced scan (coronal section). **(E)** Left internal carotid artery pseudoaneurysm (frontal view). **(F)** Left internal carotid artery pseudoaneurysm (lateral view). **(G)** Stent graft implantation (frontal view). **(H)** Stent graft implantation (lateral view).

**Figure 2:**
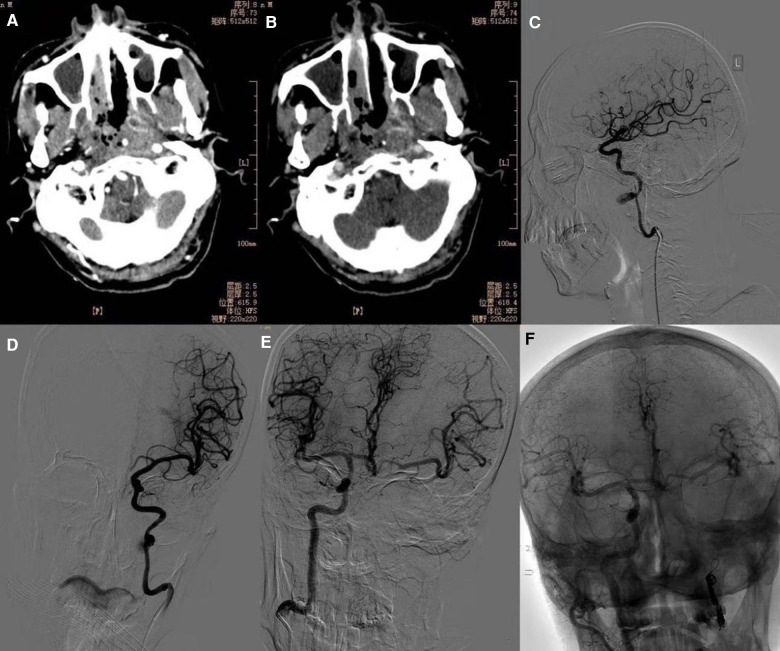
**(A,B)** The mucosa of the posterior wall of the nasopharynx is irregularly thickened, involving the bilateral parapharyngeal space. The parapharyngeal and skull base bones are partially sparse and sclerotic. **(C)** Left internal carotid artery pseudoaneurysm (lateral view). **(D)** Left internal carotid artery pseudoaneurysm (frontal view). **(E)** The balloon occlusion test showed a well-compensated left internal carotid artery. **(F)** Postoperative right internal carotid artery angiography shows well-visualized bilateral cerebral vessels.

**Figure 3:**
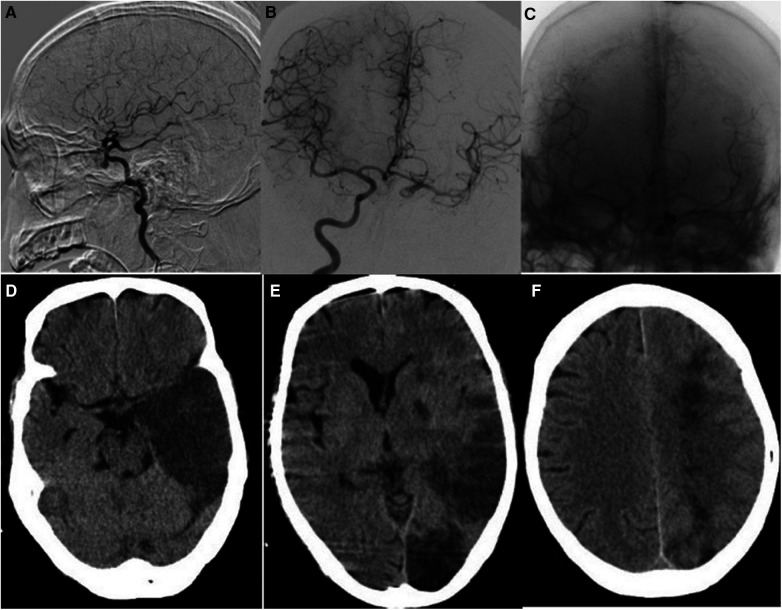
**(A)** The left internal carotid artery pseudoaneurysm in the patient ruptured. **(B)** The balloon occlusion test showed the left internal carotid artery was well compensated. **(C)** The right internal carotid angiography after left internal carotid artery coil embolization showed that the left internal carotid artery was well compensated. **(D–F)** Three days after endovascular treatment, the patient developed acute cerebral infarction in the frontotemporal lobe, occipital lobe, and left basal ganglia.

Of the 36 patients, 12 underwent stent placement (Figure 1), and 24 underwent coil occlusion (Figure 2). Postoperative bleeding was successfully controlled in all patients, achieving 100% hemostasis. Postoperative systolic blood pressure was maintained between 110 and 140 mmHg and diastolic blood pressure between 60 and 90 mmHg. One patient died 3 weeks postoperatively due to delayed bleeding after internal carotid artery stenting, and another died 2 months postoperatively due to rebleeding following internal carotid artery coil occlusion. The rate of endovascular treatment-related complications was 11.1% (4/36). Two patients who underwent internal carotid coil occlusion developed massive cerebral infarctions (Figure 3). One of these patients died, while the other survived with no severe neurological symptoms. Additionally, two patients with internal carotid artery coil occlusion experienced varying degrees of neurological symptoms: one had contralateral limb weakness with upper and lower limb strength graded as 4 and normal muscle tone, while the other experienced unilateral upper limb numbness with no abnormal muscle strength or tone. Both patients recovered within 1 week after the procedure. The 1-year survival rate following endovascular treatment was 91.7% (33/36). During the 1-year follow-up period after endovascular treatment, 11 patients underwent secondary nasal surgery, which involved the removal of necrotic tissue and flap coverage of the nasopharyngeal region.

## 4 Discussion

At present, the main treatment method for NPC is radiotherapy, often combined with chemotherapy. With the advancements in medical technology, the early diagnosis rate and 5-year survival rate have increased significantly. However, massive hemorrhage following radiotherapy for NPC remains one of the most common causes of death (8). The causes of hemorrhage after radiotherapy for NPC can be summarized as follows: (1) radiation can cause damage and necrosis of adjacent normal tissues while killing tumor tissue. The necrosis and shedding of perivascular tissue in the nasopharynx can easily expose blood vessels, and the tumor tissue may invade these vessels, leading to massive hemorrhage in the nasopharynx (9). (2) The skull can tolerate a total radiotherapy dose of about 100 Gy, while a single course of intensity-modulated radiotherapy (IMRT) typically delivers approximately 70 Gy. Repeated IMRT can exceed the skull's tolerance, potentially causing aseptic necrosis of the skull base, which can damage intraosseous and periosteal blood vessels and lead to bleeding (9). (3) NPC often occurs in the pharyngeal recess, a deep area that is prone to invading the internal carotid artery, which can result in massive hemorrhage. (4) Following radiotherapy of NPC, most of the fibers of the nasal cavity and paranasal sinuses of patients with NPC have different degrees of necrosis and fall off. This impairs the mucociliary transport system's function, leading to poor self-cleaning of the nasopharynx. Additionally, atrophy and fibrosis of the temporomandibular joint and masticatory muscles can restrict mouth opening, making the nasopharyngeal mucosa more susceptible to infection, erosion, and ulcers, which may contribute to massive bleeding (10).

Ruptured internal carotid artery pseudoaneurysms after radiotherapy for NPC can cause bleeding in the external auditory canal, nasal cavity, or oral cavity. Since the glossopharyngeal nerve is located within the radiotherapy field for NPC, patients often experience decreased pharyngeal sensation and muscle atrophy following treatment. In the event of a massive hemorrhage, patients may be unable to swallow or cough out the blood promptly. For such cases, it is crucial to maintain airway patency, with emergency tracheal intubation as needed. For acute nasal bleeding, traditional methods include nasal packing and radiofrequency cautery under nasal endoscopy. Nasal packing is simple and effective for minor nasopharyngeal bleeding. Radiofrequency cautery under nasal endoscopy offers precise and reliable hemostasis but may be inadequate for massive hemorrhage caused by rupture of internal carotid artery pseudoaneurysm. Common carotid artery or internal carotid artery ligation is the traditional treatment for carotid artery rupture syndrome. Literature reports indicate that 60% of patients develop neurological complications after surgery, with an operative mortality rate is approximately 40% (11). Surgery is particularly challenging and risky for urgent, hemodynamically unstable patients. Bypass grafting, radial artery high-flow bypass, and internal carotid artery preconditioning combined with endoscopic nasopharyngeal resection provide a new approach to the surgical treatment of nasopharyngeal carcinoma combined with ruptured pseudoaneurysm of the internal carotid artery. However, these methods may be more suitable for patients requiring limited-time surgery (5).

Occlusion of an internal carotid pseudoaneurysm using a coil or permanent balloon has a high success rate for achieving hemostasis. However, 15%–20% of patients may experience immediate or delayed neurological symptoms (12). A non-removable balloon is placed in the artery proximal to the lesion and inflated carefully until blood flow through the internal carotid artery ceases. During contralateral internal carotid artery and vertebral artery angiography, it is important to observe whether there is sufficient collateral blood flow on the ipsilateral side. Neurological examination is repeated every 3–5 min for a total test time of 20–30 min before the balloon is deflated. If the patient tolerates the test, permanent occlusion of the internal carotid artery can be considered (13). However, the balloon occlusion test may not identify patient subsets who develop delayed hemodynamic ischemia following permanent internal carotid artery occlusion. These patients may be in poor clinical condition due to ongoing profuse hemorrhage and shock. Cerebral ischemia–hypoxia may be further exacerbated in subjects undergoing balloon occlusion testing for up to 30 min. Therefore, caution is required when performing the standard balloon occlusion test in hemodynamically unstable patients. Additionally, during the radiotherapy of NPC, the contralateral internal carotid artery or vertebral artery may also be included in the radiotherapy field, which can lead to intimal changes and atherosclerosis of the contralateral internal carotid artery, resulting in insufficient blood supply.

Endovascular repair of internal carotid artery pseudoaneurysms using stent grafts reduces the incidence of neurological sequelae (14). However, there are still reports of patients experiencing cerebral infarction after stent-graft placement. Such patients are at high risk of bleeding, and the absence of antiplatelet therapy following internal carotid artery stenting may be related to thrombosis (15). Powitzky et al. (16) reported a higher risk of recurrence of internal carotid artery pseudoaneurysms with stent placement (44%) compared with embolization therapy (10%) or surgical ligation (25%). A systematic review by Bond et al. (17) involving 559 patients with carotid blowout syndrome, including the external carotid artery, revealed a rebleeding rate of 27% among all patients, 17% for those treated with coils, and 34% for those treated with covered stents. Other authors have also observed a significantly lower rate of rebleeding with embolization (11%–21%) compared with stent grafts (25%–85%) (18). Short- and long-term rebleeding after stent placement can occur from the stented carotid artery due to persistent endoluminal leakage or involvement of the carotid artery with tumor either proximal or distal to the stent and due to erosion of the arterial wall by the stent. The presence of an uncontrolled ongoing infection at the stent site is an important factor associated with recurrent internal carotid pseudoaneurysm bleeding. Therefore, some authors suggest that implanting multiple stents to extend and cover both ends of the internal carotid artery pseudoaneurysm can reduce the occurrence of endoleak (15).

For patients with unstable arterial blood flow and severe hypotension, the carotid compression test can be used as an alternative to the balloon occlusion test. This method shortens the test duration but may increase the risk of neurological dysfunction after carotid artery embolization. For patients with a positive balloon occlusion test and ineligible for stent implantation, follow-up treatment options include conservative treatment, coil embolization of the internal carotid artery, and carotid artery ligation. Conservative medical treatment is often limited in effectiveness for patients with hemodynamically unstable internal carotid artery rupture and massive hemorrhage. Many such patients may have undergone conservative treatment before interventional surgery but with poor results. Carotid ligation is rarely used due to its high surgical trauma and difficulty. Therefore, after a thorough discussion with the patient, attempting coil occlusion of the affected internal carotid artery can be considered. This approach aims to maintain hemodynamic stability during and after surgery, minimize hypotension, and reduce the incidence of neurological dysfunction symptoms as much as possible.

The author suggests that endovascular treatment of internal carotid artery pseudoaneurysm should not only involve covering both ends of the pseudoaneurysm but also include a CT examination to assess the extent of nasopharyngeal necrosis. The coil or stent must cover the internal carotid artery in the segment corresponding to the nasopharyngeal necrotic area as indicated in CT. Infected necrotic tissue in the nasopharynx that is not effectively controlled can spread along the carotid sheath; therefore, surgical removal of nasopharyngeal necrotic tissue should be performed as soon as possible after endovascular treatment to prevent the recurrence of rupture bleeding from the carotid artery pseudoaneurysm.

There are limitations in this retrospective study. (1) All patients underwent only CTA examination of cranial vessels after endovascular treatment, without additional angiography. (2) All patients were emergency cases with ruptured internal carotid artery pseudoaneurysms and were hemodynamically unstable, which may reduce the reliability of the balloon occlusion test. (3) Throughout the study period, different types of covered stents were used.

## 5 Conclusion

This retrospective analysis shows that endovascular therapy provides strong evidence as an effective and relatively safe option for managing ruptured carotid pseudoaneurysms in patients with internal carotid arteries after radiotherapy for NPC, highlighting significant survival benefits while pointing to areas where clinical practice can be improved to reduce complications.

## Data Availability

The raw data supporting the conclusions of this article will be made available by the authors, without undue reservation.
